# Metered Dose Inhaler Technique: A Priority Catch for Physicians

**DOI:** 10.7759/cureus.10857

**Published:** 2020-10-08

**Authors:** Muhammad Ahmed, Muqadas Munir, Ali Sufyan, Jahanzeb Ayyaz, Imran Arshad, Mujtaba Bukhari, Muhammad Umar, Muhammad Khurram, Ahsan Tariq, Muhammad Hamza

**Affiliations:** 1 Department of Medicine, Rawalpindi Medical University, Rawalpindi, PAK; 2 Department of Medicine, Benazir Bhutto Hospital, Rawalpindi, PAK; 3 Gastroenterology, Rawalpindi Medical University, Rawalpindi, PAK; 4 Department of Medicine, Holy Family Hospital/Rawalpindi Medical University, Rawalpindi, PAK; 5 Department of General Surgery, Benazir Bhutto Hospital, Rawalpindi, PAK

**Keywords:** asthma, inhalational technique, inhaler administration checklist, metered dose inhalers, rawalpindi, pakistan

## Abstract

Background: Asthma is a non-curable but preventable disease that can be controlled by a proper approach. Inhalational route is considered to be one of the fastest, non-invasive course for the management of asthma. Despite its importance, compliance towards proper inhalational technique remains quite low. Thus, United Kingdom guidelines and Global Strategy for Asthma Management and Prevention (GINA) recommend regular assessment of inhaler techniques in all asthma patients.

Objective: To evaluate the inhalational technique of asthma patients visiting out-patient departments of public sector tertiary care hospitals of Rawalpindi and correlate with various demographic factors.

Methods: A cross-sectional study was conducted on a total of 209 respondents visiting the outpatient department of public sector hospitals in Rawalpindi. Asthmatic patients were included via a non-probability consecutive sampling technique and were assessed for inhaler techniques via a structured checklist. Statistical data were analyzed using IBM Statistical Package for Social Sciences (SPSS®), version 25.0 (IBM Corp., Armonk, NY, USA).

Results: Two hundred and nine asthma patients were included. Only 10% of patients demonstrated the correct inhaler technique. Continuing inhaling till lungs are full, holding breath for five to 10 seconds, and breathing out slowly after using the inhaler were most poorly followed.

Conclusion: Most asthma patients are using poor inhalation technique, risking sub-optimal drug delivery and inadequate effects. Hence, it is the need of the hour to focus on patient training and education.

## Introduction

Asthma is an inflammation of the airways so its treatment comprises medications given via inhalational route [1]. Inhalational route is considered to be one of the fastest, non-invasive courses for the treatment of asthma [2]. The most common inhalational devices are metered dose inhalers (MDI), dry powdered inhalers (DPI), and nebulizers [3]. Improper inhalational technique is one of the major causes of poor asthmatic control because it leads to inadequate delivery of the drug to the respiratory tract, causing poor control of asthma [4]. Over the past 30 years, incorrect use of inhalational devices and its consequences have been documented [5]. The most commonly known mistakes associated with inhalational techniques include failure to exhale before activation, inadequate breath-holding after inhalation, improper positioning of inhalational device, wrong rotation sequence, and improper execution of forceful and deep inhalation [6].

The United Kingdom guidelines and Global Strategy for Asthma Management and Prevention (GINA) recommend that inhaler techniques should be regularly assessed in all asthma patients [7]. A study in Nigeria found more use of MDIs as compared to other inhalational techniques. According to this study, a patient’s education directly influences the inhalational technique [8]. A study conducted in Oman showed good asthmatic control to be dependent upon good inhaler techniques [9]. A Netherlands based study showed that as compared to DPIs, MDIs, Rota haler, and Trubuhaler lead to more inhalational errors [10]. A univariate analysis conducted in the USA showed better adherence to inhalational drugs among DPI users [11]. A study conducted in New Delhi, India showed maximum mistakes while using inhalational devices were committed by self-educated patients followed by those trained by pharmacists and health care professionals. Moreover, it also depicted that MDIs and nebulizers are associated with more errors as compared to DPIs [12]. A study of Civil Hospital Karachi showed more usage of MDIs by asthma patients followed by DPIs [13]. A Pakistan-based study demonstrated an inverse relationship between the education level of patients and incorrect inhaler technique [14].

The rationale of this study is to evaluate the inhalational techniques of asthma patients visiting outpatient departments of a public sector tertiary care setting in Rawalpindi, Pakistan; to assess common mistakes performed while using inhalational devices, and to discuss insinuation for clinical benefit of asthma patients. Different factors influencing inhalational techniques including patient’s age, sex, education level, and training regarding inhaler use will be assessed. This study will help in devising new strategies in treatment plans and patients’ education for better control of asthma.

## Materials and methods

This cross-sectional study was conducted at a public sector tertiary care setting after approval from the Institutional Research Forum. Using the World Health Organization (WHO) sample size calculator with 95% confidence level, 5% margin of error, and 16.3% estimated prevalence the sample size was calculated to be 209 [14].

Patients were included via a non-probability consecutive sampling technique. Patients of age greater than 12 years diagnosed with asthma and using MDIs as inhalers were included in the present study. Patients using inhalers others than MDIs or diagnosed on the same visit were excluded from the study. The procedure was explained individually to the patients and prior verbal consent was taken. Using structured proforma, demographic data including patient gender, age, educational status, and economic status was collected from patients. Data regarding the duration of use and place of first prescription was also taken. The inhaler technique was assessed using a 10-step standard checklist adopted from American guidelines.

Data was entered on Intel Business Machine Statistical Package for Social Sciences (SPSS) version 25 (IBM Corp., Armonk, NY, USA). Descriptive analysis was done on quantitative variables while frequency tables were applied to assess qualitative variables. The patient's inhalational technique was considered correct if eight or more out of ten steps were correct according to the checklist. Pearson’s correlation was used to determine the statistical significance of factors affecting inhalational technique.

## Results

Out of a total of 209, 56.5% (n=118) were male and 43.5% (n=91) were female. Mean age of patients was 37.4 ± 22.1 years. Patient characteristics are given in Table 1.

**Table 1:** Characteristics of studied patients SD: standard deviation

Variable	N (%)
Gender	
Male	118 (56.5)
Female	91 (43.5)
Age (years)	
Mean (SD)	37.4 (±22.1)
Educational Status	
No formal education	111 (53.1)
Literate	98 (46.9)
Economic Status	
Lower	145 (69.4)
Middle	64 (30.6)
Place of first prescription	
Tertiary care	145 (69.4)
Non Tertiary care	64 (30.6)
Duration of use	
24 months or less	80 (38.3)
More than 24 months	129 (61.7)

**Figure 1:**
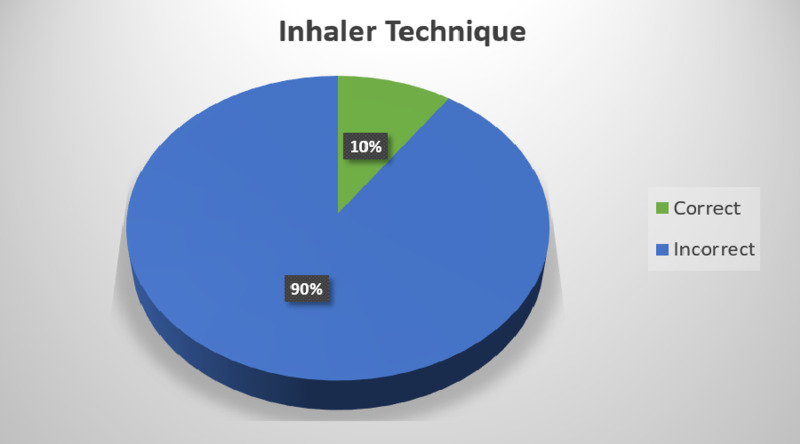
Pie chart representing percentage of patients using correct inhaler technique.

Only 10% (n=20) used the inhaler with correct technique while 90% (n=209) had an incorrect technique (Figure 1).

Pearson’s chi-square was applied to see the association of gender, educational status, economic status, place of first prescription and duration of use with inhaler technique. Females were more likely to use the incorrect technique (x^2^(1)=0.113 p=0.737). Patients with no formal education were also more likely to use an incorrect technique (x^2^(1)=0.086 p=0.769). Patients with the lower middle class and those who were prescribed inhalers at tertiary care settings were more likely to use the incorrect technique (x^2^(1)=0.200 p=0.655, x^2^(1)=1.175 p=0.278, respectively). Patients using the inhaler for more than 24 months were also more likely to use the incorrect technique (x^2^(1)=1.286 p=0.257), however, these associations were not significant (Table 2).

**Table 2:** Comparison of inhaler technique according to characteristics of patients

Characteristic	Correct Technique N (%)	Incorrect Technique N (%)	Total N (%)	x^2^ (P value)*
Gender	20 (10)	189 (90)	209 (100)	0.113 (0.737)
Male	12 (10.2)	106 (89.8)	118	
Female	8 (8.8)	83 (91.2)	91	
Educational Status	20 (10)	189 (90)	209 (100)	0.086 (0.769)
No formal education	10 (9)	101 (91)	111	
Literate	10 (10.2)	88 (89.8)	98	
Economic Status	20 (10)	189(90)	209 (100)	0.200 (0.655)
Lower	13 (9)	132 (91)	145	
Middle	7 (10.9)	57 (89.1)	64	
Place of first prescription	20 (10)	189 (90)	209 (100)	1.175 (0.278)
Tertiary care	16 (11)	129 (89)	145	
Non Tertiary care	4 (6.3)	60 (93.8)	64	
Duration of use	20 (10)	189 (90)	209 (100)	1.287 (0.257)
24 months or less	10 (12.5)	70 (87.5)	80	
More than 24 months	10 (7.8)	119 (92.2)	129	
*Chi-Square was applied to calculate P value				

Out of 10 steps of inhaler technique according to American guidelines, the most-followed steps were to take the cap off the inhaler, hold the inhaler upright, place the mouthpiece between lips, and trigger the inhaler. The least-followed step was to hold the breath for five to 10 seconds - followed by only 15% of patients.

## Discussion

This hospital-based observational study of 209 asthma patients showed that the majority of patients are using incorrect inhaler techniques. The study showed that only 10% of asthma patients using MDIs have correct inhaler techniques and were able to complete all steps as per American guidelines, however, a similar study conducted in asthma patients in Nigeria showed that 22.1% of patients practice correct inhaler technique [8]. Similarly, van Beereendonk et al. in the Netherlands showed that 11.1% of asthma patients complete the required steps of correct inhaler technique [15]. Among a total of 10 steps of correct inhaler technique as per American guidelines, our study showed that the least-practiced step is holding the breath while counting to 10, which is practiced appropriately by only 7.6% of the total study population; however, an American study showed that about 50% of asthma patients practice this step while using inhalers [11]. The most common error in the use of MDI in our study was step 8, holding the breath for five to 10 seconds, while the study in Nigeria and other studies identified step 7, continuing to inhale until lungs are full, as the most common mistake made by MDI users [10,16].

Our study showed that illiterate asthma patients are more likely to use incorrect inhaler technique as compared to literate patients. Patients having formal education have comparatively better inhaler technique than those with no education; however, tests of significance didn’t show a significant association between educational status and inhaler technique. Our result is similar to a study conducted in Karachi which showed that patients with no education and under matriculate group had incorrect inhaler technique [14]. The result is also comparable to the study conducted in Nigeria which showed only a few asthma patients with no formal or with primary education completed the steps of inhaler technique than those with secondary and tertiary education [8]. Therefore, educational status and inhaler technique have an inverse relation to each other.

The present study showed a non-significant association between female gender and incorrect inhaler technique; females were found to be more likely to use an incorrect inhaler technique than males. Similarly, Goodman et al. observed the inhalational technique of males to be better than females [17]. On the contrary, Gray et al. found no significant association between sex and incorrect MDI inhaler technique among asthma patients [18]. Our study concluded that patients belonging to the lower middle class have more incorrect inhaler techniques than upper-middle and higher class, however Pearson’s chi-square test did not show a significant association between economic status and correct inhaler technique. This result is comparable to a study conducted in New Delhi, India, which showed that maximum errors in inhaler technique were made by patients belonging to low economic status [12]. Patients prescribed inhalers at tertiary care settings are more likely to use incorrect technique, however, this association was not significant. Our study showed that patients using the inhaler for more than 24 months are more likely to use incorrect technique than those using less than 24 months. This is similar to a study in Saudi Arabia which stated that patients diagnosed with asthma for less than 24 months used inhaler improperly compared to those diagnosed for more than 24 months [19].

The present study is not without limitations; it could not be assessed whether the patients were taught the correct inhalational technique by the health care professionals in the first place. A Brazil-based study has highlighted how a majority of health care professionals were unable to demonstrate adequate technique [20]. Hence, the knowledge of health care professionals and effective communication might be playing a crucial role in determining the adequate inhalational technique. Additionally, the patient’s education on compliance, importance of adequate inhalational technique, and poor mini-mental state examination might be some other significant factors [8,10]. Aside from studying the determinants of inhaler technique, an insight on its outcomes appears to be equally concerning. Improper inhalation technique and poor compliance have been independently linked with achieving poor asthma controls [9,21].

This study forms the basis to generate future work in this novel area, including assessment of health-care providers knowledge of correct inhaler technique. It will equally form the basis of continued education of the patients who use these inhalers during their routine hospital visits. In order to ensure optimal drug delivery and subsequently achieve asthma control, it is of utmost importance to devise effective patient-directed educational strategies and regular assessments of patient compliance.

## Conclusions

Despite the availability of adequate guidelines and manuals, most of the asthma patients were using poor inhalational techniques. Prolonged usage and prescription from a tertiary care setup were a risk factor for poor inhalational technique. Patients who were illiterate and those who belonged to poor socioeconomic status were found to be at higher risk of using poor inhaler technique. Hence, it is imperative to focus on patient training and education as it is putting patients at risk for not achieving optimal asthma control and thereby losing compliance.
